# Systemic Inflammatory and Hematological Profiles in Triple-Negative Breast Cancer: A Study from a Senegalese Cohort

**DOI:** 10.3390/diagnostics16030494

**Published:** 2026-02-06

**Authors:** Nènè Oumou Kesso Barry, Mamadou Sow, Pape Matar Kandji, Ndeye Khady Ngom, Moustapha Djité, Mouhamad Sy, Salif Baldé, Ulrich Igor Mbessoh Kengne, Amacoumba Fall, Siny Ndiaye, Ndeye Marème Thioune, Jaafar Thiam, Amadi Amadou Sow, Fidèle Kiema, Cheikh Tidiane Gassama, Simbi Celestin Kitungwga, Yacine Mbacke, Marième Guetti, Marie Masesi Lusasi, Fatou Gueye Tall, El Hadj Malick Ndour, Amy Gaye, Aboubacar Dit Tietie Bissan, Mariama Touré, Aïta Sène, Assiatou Barry, Saikou Oumar Diallo, Dominique Doupa, Najah Fatou Coly, Cherif Dial, Ahmadou Dem, Sidy Ka, Pascal Reynier, Papa Madieye Gueye

**Affiliations:** 1 Pharmaceutical Biochemistry Laboratory, Cheikh Anta Diop University, Dakar BP 5005, Senegal; 2 Biochemistry Laboratory, University Hospital Center of Fann, Dakar BP 5035, Senegal; 3 Oncology Department, Dalal Jamm Hospital, Dakar B.P.19001, Senegal; 4 Pathology Laboratory, Cheikh Anta Diop University, Dakar BP 5035, Senegal; 5 International Center for Research and Training in Applied Genomics and Health Surveillance (CIGASS), Dakar BP 16477, Senegal; 6 Faculty of Pharmacy, Medicine and Odontostomatology, University of Sciences, Techniques and Technologies of Bamako (USTTB), Bamako BP E423, Mali; 7 Department of Medical Biochemistry, Saint-Louis University, Saint-Louis BP 234, Senegal; 8 Medical Biology Laboratory, Diamniadio Children’s Hospital, Dakar BP 204, Senegal; 9 Department of Biochemistry and Molecular Biology, University Hospital of Angers, 49933 Angers, France

**Keywords:** triple-negative breast cancer (TNBC), hematological parameters, neutrophil-to-lymphocyte ratio (NLR), monocyte-to-lymphocyte ratio (MLR), C-reactive protein (CRP), breast cancer subtypes, systemic inflammation

## Abstract

**Background/Objectives**: Triple-negative breast cancer (TNBC) is an aggressive subtype associated with a poor prognosis and limited treatment options. Inflammatory and hematological biomarkers have emerged as potential tools for disease characterization, particularly in low-resource settings. **Methods**: This cross-sectional analytical study was conducted between July 2022 and February 2024 at Dalal Jamm Hospital in Dakar, Senegal, and included 120 women: 40 with TNBC, 40 with hormone-dependent breast cancer (HDBC), and 40 healthy controls. Blood samples were collected at diagnosis before any treatment to measure complete blood counts and C-reactive protein (CRP) levels. Inflammatory ratios—neutrophil-to-lymphocyte ratio (NLR), monocyte-to-lymphocyte ratio (MLR), and platelet-to-lymphocyte ratio (PLR)—were calculated. **Results**: TNBC patients displayed a distinct inflammatory profile characterized by elevated neutrophil counts, CRP, NLR, and MLR, as well as reduced lymphocyte and basophil percentages compared to healthy controls. NLR > 1.12 demonstrated strong discriminatory ability (AUC = 0.847; sensitivity 90%; specificity 65%). Differences between TNBC and HDBC were less pronounced, except for CRP and basophil levels. Multivariate analysis confirmed independent associations of elevated NLR, CRP, and neutrophils with TNBC. **Conclusions**: These findings provide new insights into the inflammatory and hematological characteristics of TNBC in this population and support further investigation of accessible biomarkers for early disease stratification in similar settings.

## 1. Introduction

Breast cancer is the most diagnosed cancer and the leading cause of cancer-related death among women worldwide, with over 2.3 million new cases and 685,000 deaths annually [1]. Among its subtypes, triple-negative breast cancer (TNBC)—defined by the absence of estrogen receptor (ER), progesterone receptor (PR), and HER2 expression—accounts for 10–20% of cases and is associated with an aggressive clinical course, early recurrence, and limited treatment options compared to hormone-dependent breast cancer (HDBC) [1,2].

Systemic inflammation has emerged as a key factor in cancer development, progression, and treatment response. Several hematologic markers derived from routine blood tests—such as the neutrophil-to-lymphocyte ratio (NLR), platelet-to-lymphocyte ratio (PLR), and monocyte-to-lymphocyte ratio (MLR)—are recognized as indicators of systemic inflammation. C-reactive protein (CRP), an acute-phase reactant induced by interleukin-6 and other cytokines, is also a sensitive marker of inflammation [3,4,5].

Neutrophils contribute to tumor progression through immunosuppression, reactive oxygen species (ROS) production, and pre-metastatic niche formation [6]. Elevated neutrophil counts and increased NLR have been associated with poor prognosis across multiple cancers, including breast cancer [7,8,9]. Platelets support tumor growth by protecting circulating tumor cells and promoting angiogenesis [10]. High NLR and PLR are both linked to poor outcomes, while elevated CRP levels correlate with impaired immune function and worse prognosis [11,12,13,14]. Monocytes, through differentiation into tumor-associated macrophages, also play a role in tumor immune evasion, positioning MLR as another relevant marker [5].

However, most studies have focused on the prognostic value of these markers in patients already undergoing treatment [15,16]. Few have assessed their diagnostic utility at baseline, particularly in therapy-naïve patients. While some evidence supports their ability to distinguish malignant from benign lesions [7], direct comparisons between TNBC, HDBC, and healthy individuals remain rare—especially in African populations, where genetic and environmental differences may influence disease biology.

This study aimed to characterize the hematologic and inflammatory profiles of treatment-naïve breast cancer patients in Senegal, comparing TNBC and HDBC with healthy controls. The goal was to identify inflammatory markers that could assist in the early biological characterization of TNBC in low-resource settings.

## 2. Materials and Methods

### 2.1. Ethical Approval

The study was approved by the National Ethics Committee for Health Research of Senegal (CNERS) under reference number 0000309/MSAS/CNERS/SP (approval date: 1 December 2022). Written informed consent was obtained from all participants.

### 2.2. Study Design and Participants

This prospective, cross-sectional, and analytical study was conducted from July 2022 to February 2024 at the Oncology Department of Dalal Jamm Hospital, Dakar, Senegal. The study included 120 participants: 40 with triple-negative breast cancer (TNBC), 40 with hormone-dependent breast cancer (HDBC), and 40 healthy controls.

### 2.3. Recruitment of Breast Cancer Patients

Preselection Phase: Women aged ≥ 18 years with clinical suspicion of breast cancer based on BI-RADS (Breast Imaging Reporting and Data System) categories 3, 4, or 5 were eligible if they were not pregnant, had no prior cancer treatment, and provided written informed consent.

Final Inclusion: Only patients with histologically confirmed invasive carcinoma were included. Molecular classification was based on immunohistochemistry (IHC):

- TNBC Group: ER-negative, PR-negative, and HER2-negative (IHC 0 or 1+).
- HDBC Group: ER-positive and/or PR-positive, and HER2-negative (IHC 0 or 1+).

### 2.4. Recruitment of Healthy Controls

Healthy controls were recruited during organized breast cancer screening campaigns. These participants were age-matched to cases within ±2 years and met the following inclusion criteria: (1) normal mammography (BI-RADS category 1), (2) no personal history of cancer, (3) absence of acute or chronic systemic diseases, and (4) documented written informed consent obtained prior to enrollment.

### 2.5. Blood Collection and Laboratory Analysis

Peripheral blood was collected from all participants after an overnight fast. Samples were drawn into two tubes: one with K3EDTA for complete blood count (CBC) and another with clot activator for C-reactive protein (CRP) analysis.

CBCs were performed the same day at CHU Fann (Dakar) using a Sysmex XN-1000 analyzer (Sysmex, Kobe, Japan), providing absolute counts of neutrophils, lymphocytes, monocytes, and platelets. These values were used to calculate the neutrophil-to-lymphocyte ratio (NLR), platelet-to-lymphocyte ratio (PLR), and monocyte-to-lymphocyte ratio (MLR).

CRP samples were processed at the CIGASS Biobank. After centrifugation at 4 °C (3000× *g*, 10 min), serum was aliquoted and stored at −80 °C. CRP levels were measured using immunoturbidimetry (ARCHITECT ci4100, Abbott Diagnostics, Abbott Park, IL, USA). All samples were anonymized according to biobanking and ethical standards.

### 2.6. Histological and Immunohistochemical Analysis

Breast tissue samples were fixed in formalin, paraffin-embedded, and sectioned (3 µm). Hematoxylin and eosin (H&E) staining confirmed the cancer diagnosis. Immunohistochemistry (IHC) was performed using Quartett^®^ RTU monoclonal antibodies (Quartett Immunodiagnostika & Biotechnologie GmbH, Berlin, Germany). for ER, PR, HER2, and Ki-67, and detected via PolyQ Stain 1 Step HRP/DAB system (Quartett Immunodiagnostika & Biotechnologie GmbH, Berlin, Germany). Slides were counterstained with hematoxylin and examined under light microscopy. Interpretation followed ASCO/CAP guidelines [17]. ER and PR positivity was defined as ≥1% nuclear staining. HER2 expression: 3+ = positive; 0–1+ = negative; 2+ = equivocal (requiring ISH confirmation).

### 2.7. Definition of Hematological Abnormalities

Anemia was defined as hemoglobin <12 g/dL. Platelet anomalies included thrombocytosis (>400 G/L) and thrombocytopenia (<150 G/L). White blood cell (WBC) abnormalities included hyperleukocytosis (>10 G/L) and leukopenia (<4 G/L); neutropenia and neutrophilia were defined as <2 G/L and >7.5 G/L, respectively; lymphocytosis: >4 G/L; lymphopenia: <1 G/L; monocytosis: >1 G/L; monocytopenia: <0.2 G/L.

### 2.8. Statistical Analysis

Statistical analyses were performed using SPSS version 25.0. Cut-off values for NLR, PLR, and MLR were determined by ROC analysis. Data normality was tested with the Shapiro–Wilk and Kolmogorov–Smirnov tests. Quantitative data are expressed as mean ± SD or median (IQR), and qualitative data are expressed as frequencies and percentages.

Group comparisons used repeated-measures ANOVA or the Friedman test, with Bonferroni or LSD post hoc tests. Categorical variables were analyzed using McNemar or McNemar–Bowker tests. Variables with *p* < 0.1 in bivariate analysis were included in logistic regression to estimate adjusted odds ratios (ORs) with 95% confidence intervals. A *p*-value < 0.05 was considered statistically significant.

## 3. Results

### 3.1. General Characteristics of the Study Population

We first examined the sociodemographic, anthropometric, gyneco-obstetric, and tumor characteristics of the participants to identify potential confounding or accompanying factors. The sociodemographic and clinical characteristics of the study population are summarized in Table 1. While age was comparable across groups, significant differences were noted in BMI and reproductive history. Healthy controls had a significantly higher BMI than TNBC patients (*p* = 0.003), with more frequent overweight and obesity (*p* = 0.047). TNBC patients also exhibited higher gravidity (*p* = 0.040) and parity (*p* < 0.05) compared to HDBC and controls.

No significant differences were observed for age at menarche, contraceptive use, menopausal status, or family history of cancer. A sedentary lifestyle was prevalent in all groups, while smoking and alcohol use were absent or minimal.

Regarding tumor features, both TNBC and HDBC patients presented with advanced disease. Nearly one-third of patients were diagnosed at stage IV, with no significant difference between groups (*p* = 0.975), indicating a comparable metastatic burden at diagnosis.

### 3.2. Hematological Profiles Among TNBC, HDBC, and Healthy Controls

In addition to the clinical characteristics, we assessed basic hematological parameters to determine whether significant biological differences existed between the groups. The hematological parameters of the study population are presented in Table 2. No significant differences were observed between groups for red blood cell count, hemoglobin, hematocrit, or platelet levels.

However, significant differences were observed in several red and white blood cell indices. The mean corpuscular volume (MCV) was significantly lower in the TNBC group compared to healthy controls (*p* = 0.025), as was the mean corpuscular hemoglobin (MCH) (*p* = 0.046).

White blood cell (WBC) counts were significantly higher in both cancer groups compared to healthy controls (*p* = 0.018). Neutrophil counts and neutrophil percentage were significantly elevated in the TNBC group compared to the other groups (*p* < 0.001). In contrast, lymphocyte percentages were significantly lower in cancer patients (*p* < 0.001).

Basophil counts and percentages, as well as monocyte counts, also showed statistically significant differences among groups (*p* < 0.05). Eosinophil percentages were significantly lower in TNBC and HDBC patients compared to healthy controls (*p* = 0.019).

### 3.3. Systemic Inflammatory Profile: NLR, PLR, MLR, and CRP

**Figure 1:**
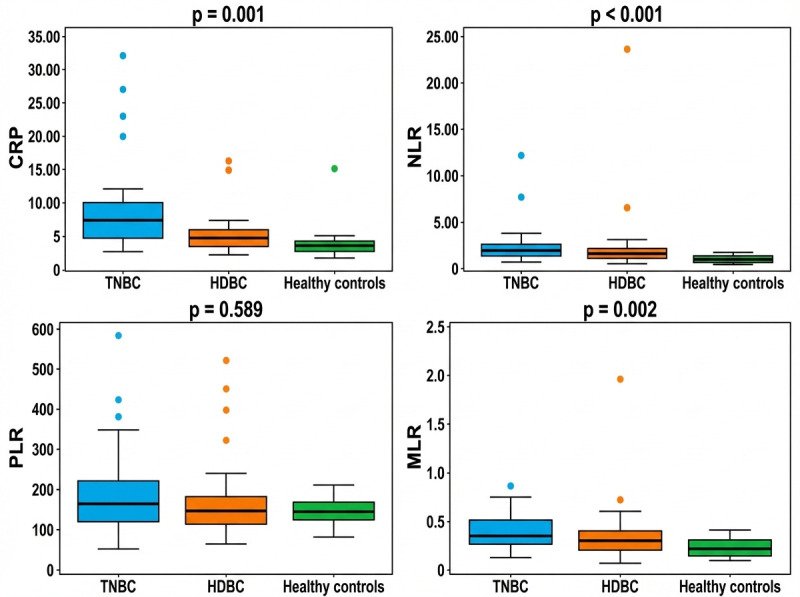
Inflammatory markers in breast cancer subtypes and controls. Abbreviations: CRP = C-reactive protein; NLR = neutrophil-to-lymphocyte ratio; PLR = platelet-to-lymphocyte ratio; MLR = monocyte-to-lymphocyte ratio; TNBC = triple-negative breast cancer; HDBC = hormone-dependent breast cancer.

Beyond standard hematological parameters, we further analyzed systemic inflammatory markers—including the neutrophil-to-lymphocyte ratio (NLR), platelet-to-lymphocyte ratio (PLR), monocyte-to-lymphocyte ratio (MLR), and C-reactive protein (CRP)—which may reflect the patients’ immune and inflammatory status. The distribution of these markers across the three groups—triple-negative breast cancer (TNBC), hormone-dependent breast cancer (HDBC), and healthy controls—is illustrated in Figure 1. The medians of CRP, NLR, and MLR were significantly higher in the TNBC and HDBC groups compared to healthy controls, with *p*-values of 0.001, 0.000, and 0.002, respectively. In contrast, the median PLR values did not differ significantly between the groups (*p* = 0.589).

### 3.4. Pairwise Comparisons: TNBC vs. HDBC and Healthy Controls

**Figure 2:**
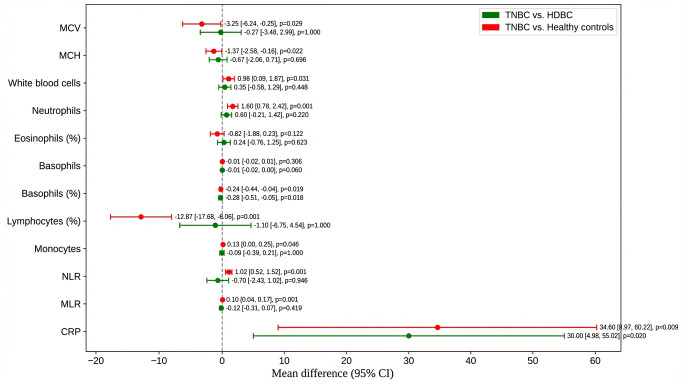
Post hoc comparisons of hematologic and inflammatory parameters. Mean differences and 95% confidence intervals are shown for parameters including NLR, CRP, MCV, and MCH, comparing TNBC to HDBC and controls. Abbreviations: TNBC = triple-negative breast cancer; HDBC = hormone-dependent breast cancer; NLR = neutrophil-to-lymphocyte ratio; CRP = C-reactive protein; MCV = mean corpuscular volume; MCH = mean corpuscular hemoglobin; CI = confidence interval.

To further explore intergroup differences, post hoc comparisons were performed between TNBC patients and both HDBC patients and healthy controls (Figure 2).

Compared to healthy controls, TNBC patients had a significantly lower mean corpuscular volume (MCV) (–3.25; *p* = 0.029) and mean corpuscular hemoglobin (MCH) (–1.37; *p* = 0.022), with no significant difference versus HDBC. TNBC patients also had higher white blood cell counts (+0.98; *p* = 0.031) and neutrophils (+1.60; *p* = 0.001) than healthy controls, but they were not significantly different from HDBC.

Lymphocyte percentage was markedly reduced in TNBC versus healthy controls (–12.87; *p* = 0.001); basophil percentage was lower in TNBC compared to both HDBC (*p* = 0.018) and controls (*p* = 0.019). Monocyte count was slightly higher in TNBC versus controls (+0.13; *p* = 0.046).

Regarding inflammatory markers, NLR and MLR were significantly elevated in TNBC versus healthy controls (*p* = 0.001 and *p* = 0.001, respectively), but not versus HDBC. CRP levels were significantly higher in TNBC compared to both HDBC (*p* = 0.020) and healthy controls (*p* = 0.009).

### 3.5. ROC-Based Evaluation of Inflammatory Ratios Between Groups

**Figure 3:**
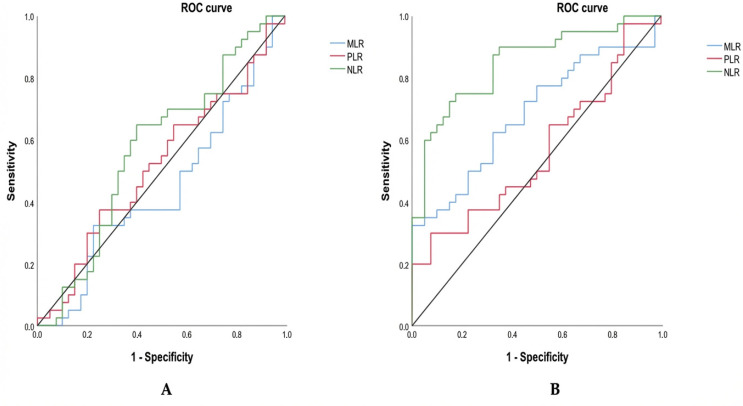
ROC curves for inflammatory markers distinguishing TNBC. Receiver operating characteristic (ROC) curves compare NLR, MLR, and PLR in distinguishing TNBC from (**A**) HDBC and (**B**) healthy controls. Abbreviations: TNBC = triple-negative breast cancer; HDBC = hormone-dependent breast cancer; NLR = neutrophil-to-lymphocyte ratio; PLR = platelet-to-lymphocyte ratio; MLR = monocyte-to-lymphocyte ratio.

To assess the diagnostic value of these inflammatory markers, we subsequently performed a receiver operating characteristic (ROC) analysis to evaluate their ability to discriminate between groups. The ROC-based evaluation of inflammatory ratios across groups shows contrasting diagnostic performance depending on the comparison (Table 3; Figure 3). When comparing triple-negative breast cancer (TNBC) to hormone-dependent breast cancer (HDBC) (Figure 3A), none of the markers—NLR (cut-off: 1.55), MLR (cut-off: 0.24), or PLR (cut-off: 144.73)—demonstrated statistically significant discrimination. This is reflected by AUC values close to 0.5 and non-significant *p*-values (all *p* > 0.05), indicating limited diagnostic utility. Among them, NLR had the highest AUC (0.572), but it remained non-significant (*p* = 0.268).

In contrast, when TNBC patients were compared to healthy controls (Figure 3B), both NLR and MLR demonstrated significant discriminatory capacity. As presented in Table 3 and illustrated in Figure 2, NLR (cut-off: 1.12) displayed a high ability to distinguish between the two groups, with an AUC of 0.847, a sensitivity of 90%, a specificity of 65%, and a highly significant *p*-value (*p* = 0.000). MLR (cut-off: 0.20) also showed moderate diagnostic accuracy, with an AUC of 0.684 (sensitivity 62.5%, specificity 67.5%, *p* = 0.005). Conversely, PLR (cut-off: 136.50) did not reach statistical significance in this comparison either (AUC = 0.563, *p* = 0.331), confirming its limited value for distinguishing TNBC from healthy individuals.

### 3.6. Frequency of Hematological Alterations and Elevated Inflammatory Markers

**Figure 4:**
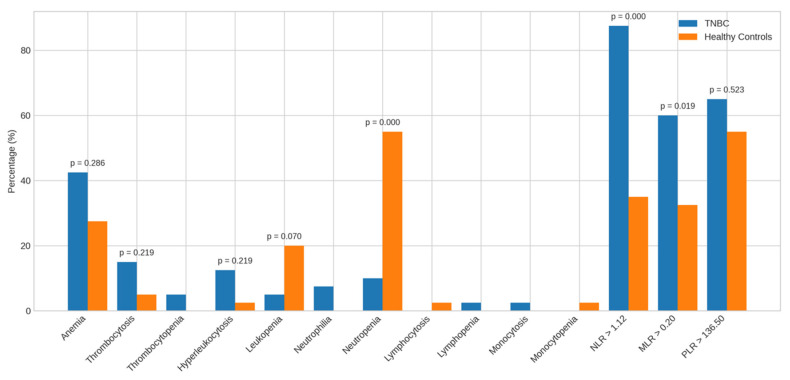
Alterations in the parameters of TNBC patients vs. healthy controls. Bar charts display the frequency of abnormal hematological and inflammatory markers among TNBC patients compared to healthy controls. Abbreviations: TNBC = triple-negative breast cancer.

**Figure 5:**
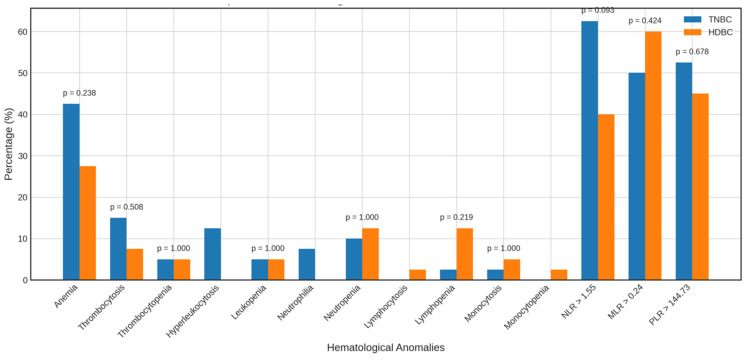
Alterations in the parameters of TNBC vs. HDBC patients. The figure compares the prevalence of hematologic and inflammatory abnormalities in TNBC and HDBC patient groups. Abbreviations: TNBC = triple-negative breast cancer; HDBC = hormone-dependent breast cancer.

Beyond mean and median values, we analyzed the frequency of hematological abnormalities and elevated inflammatory markers to better characterize TNBC profiles (Figure 4 and Figure 5).

Compared to healthy controls, TNBC patients showed no significant differences in the prevalence of anemia (42.5% vs. 27.5%), thrombocytosis (15.0% vs. 5.0%), leukocytosis (12.5% vs. 2.5%), or leukopenia (5.0% vs. 20.0%). However, neutropenia was significantly more frequent in controls (55.0%) than in TNBC patients (10.0%, *p* = 0.000). Other anomalies were observed but not statistically analyzed due to low frequency.

A higher proportion of TNBC patients had elevated NLR > 1.12 (87.5% vs. 35.0%, *p* = 0.000) and MLR > 0.20 (60.0% vs. 32.5%, *p* = 0.019) than controls; PLR > 136.50 did not differ significantly.

When compared to HDBC, TNBC patients showed no significant differences in anemia (42.5% vs. 27.5%), thrombocytosis, or other hematologic abnormalities. A non-significant trend toward a higher NLR > 1.55 was observed in TNBC (62.5% vs. 40.0%, *p* = 0.093), with no meaningful differences for MLR > 0.24 or PLR > 144.73.

### 3.7. Multivariate Associations of Hematological and Inflammatory Markers in TNBC Versus HDBC and Healthy Controls

To determine factors independently associated with triple-negative breast cancer (TNBC), we conducted conditional logistic regression models using variables with *p* < 0.1 in the univariate analysis (Table 4). Given the relatively small sample size, we applied a deliberately parsimonious strategy to minimize the risk of overparameterization and limit model complexity. In each model, a maximum of four biologically coherent explanatory variables were included, consistent with the classical events-per-variable rule (EPV ≥ 10) and the balanced design of the study (40 subjects per group).

Compared to hormone-dependent breast cancer (HDBC), TNBC was independently associated with higher neutrophil counts (adjusted OR = 1.61; *p* = 0.034), presence of inflammation (OR = 5.00; *p* = 0.011), and elevated NLR > 1.55 (OR = 5.08; *p* = 0.013). In contrast, lower mean corpuscular hemoglobin (MCH) (OR = 0.62; *p* = 0.036), reduced basophil percentage (OR = 0.30; *p* = 0.035), and MLR > 0.24 (OR = 0.21; *p* = 0.038) were inversely associated.

When compared to healthy controls, TNBC was associated with higher neutrophil counts (OR = 12.21; *p* = 0.007), presence of inflammation (OR = 4.92; *p* = 0.010), elevated NLR (OR = 26.43; *p* = 0.002), and NLR > 1.12 (OR = 11.5; *p* = 0.001), along with lower MCH, lymphocyte percentage, total WBC count, and neutropenia (all *p* < 0.05). CRP showed a modest association (OR = 1.02; *p* = 0.026), while PLR did not reach statistical significance (*p* = 0.053).

Taken together, these findings highlight a distinct inflammatory and hematological profile in patients with TNBC. These observations are discussed further in the following section, considering the existing literature.

## 4. Discussion

This study aimed to identify accessible hematological and inflammatory biomarkers associated with triple-negative breast cancer (TNBC) in a Senegalese cohort. TNBC patients exhibited a distinct clinical and biological profile, consistent with its aggressive and immunologically active nature.

Although age did not differ significantly between groups, the younger average age of TNBC patients (46 years) aligns with previous findings in Senegal [18,19] and Ghana [20]. This finding is consistent with the known tendency of TNBC to occur at younger ages, including in Western populations [21]. Therefore, the observed age may not substantially differ from TNBC cohorts in high-income countries. However, this earlier onset compared to breast cancer overall may still reflect genetic or environmental factors associated with African ancestry [22].

The lower BMI observed in TNBC patients versus healthy controls may suggest either cancer-related cachexia or delayed diagnosis, although some studies report a complex relationship between BMI and TNBC, particularly in premenopausal women [21,23].

Hematologically, TNBC patients showed elevated neutrophils and reduced lymphocyte percentages, indicating systemic inflammation and immunosuppression, consistent with neutrophil-driven tumor mechanisms [24]. Increased monocytes and WBCs further support immune activation and potential TAM (tumor-associated macrophage) involvement [24]. This inflammatory profile may be particularly relevant in the context of predominantly advanced-stage disease, where chronic systemic inflammation contributes to tumor progression, immune evasion, and T-cell exhaustion. Elevated neutrophils can promote tumor growth and metastasis through the release of pro-tumorigenic factors (e.g., VEGF, MMPs), while reduced lymphocytes reflect impaired anti-tumor immunity. Monocytosis may reflect the recruitment of monocytes to the tumor microenvironment, where they differentiate into TAMs that support tumor survival and suppress adaptive immune responses. Together, these findings suggest an immune landscape shaped by both inflammation and immune dysregulation, characteristic of aggressive TNBC biology in advanced disease stages.

Lower MCV and MCH suggest anemia of chronic disease, associated with disrupted iron metabolism and bone marrow suppression [25].

Inflammatory markers—CRP, NLR, and MLR—were significantly higher in TNBC and HDBC patients compared to controls, reinforcing their role in breast cancer pathophysiology. Elevated CRP correlates with tumor burden and poor prognosis [26], while high NLR reflects neutrophil-driven immune suppression and is linked to poor outcomes, particularly in TNBC [12,27,28]. MLR elevation suggests monocyte involvement in tumor progression [29]. PLR, however, showed no significant difference, aligning with studies that suggest its prognostic rather than diagnostic relevance [30,31,32].

This may reflect the fact that, unlike neutrophils and monocytes, both of which are directly involved in tumor-promoting inflammation and immune suppression, platelets play a more indirect role in cancer progression, such as by facilitating angiogenesis and shielding circulating tumor cells. In contrast, NLR and MLR reflect more direct immune alterations commonly observed in aggressive tumors like TNBC, namely neutrophilia, lymphopenia, and monocytosis, making them more sensitive indicators of tumor-associated inflammation and immune dysregulation. Therefore, while PLR may retain prognostic usefulness, it appears less informative for diagnostic discrimination in this cohort.

Pairwise comparisons highlighted lower MCV and MCH, higher neutrophils and WBCs, and reduced lymphocytes in TNBC versus controls, reflecting inflammation and immune exhaustion [33,34,35]. Although NLR was not significantly different between cancer subtypes, CRP was more elevated in TNBC, supporting its subtype-specific role [34,35]. Elevated MLR in TNBC versus controls also indicated monocyte involvement, though differences with HDBC were not significant.

Notably, basophil percentages were lower in TNBC than in both comparison groups, possibly reflecting impaired immune surveillance [36].

As shown in the ROC analysis, NLR and MLR were able to differentiate TNBC patients from healthy controls, with NLR showing the highest area under the curve (AUC = 0.847; cut-off = 1.12; sensitivity = 90%; specificity = 65%). MLR showed moderate separation (AUC = 0.684; *p* = 0.005), while PLR had limited ability to differentiate groups (AUC = 0.563; *p* = 0.331). These findings suggest that NLR may reflect a systemic inflammatory state associated with the presence of cancer. Similar trends have been reported in previous studies, where NLR, PLR, and CRP showed good capacity to distinguish benign from malignant breast lesions [7]. Moreover, elevated NLR (>2.65) and PLR (>190.9) have been associated with worse 5-year overall survival, especially in ER-negative and TNBC cases, reinforcing their potential prognostic relevance [5]. Conversely, MLR has shown limited prognostic value in other studies [5].

However, in comparisons between TNBC and HDBC, the ROC analysis revealed that AUCs for NLR, MLR, and PLR were close to 0.5 and not statistically significant, indicating that these markers lack the ability to distinguish between breast cancer subtypes. This suggests that the inflammatory alterations captured by these markers are more reflective of general cancer-associated immune activation than of subtype-specific immune signatures.

Multivariate analysis confirmed that high neutrophil counts, CRP, and inflammation were independently associated with TNBC versus both HDBC and controls. NLR remained a consistent marker at both 1.12 and 1.55 thresholds. Lower MCH and lymphocyte percentages were also independently associated with TNBC, supporting the link to chronic inflammation and immune dysfunction [6,37]. Interestingly, MLR > 0.24 was inversely associated with TNBC vs. HDBC, possibly reflecting immune differences. The inverse association with neutropenia and WBC count in TNBC versus controls suggests excessive neutrophil activation—a hallmark of aggressive inflammation.

The categorical analysis of hematological abnormalities added further support. Though most standard abnormalities were not significant, inflammatory ratios such as NLR > 1.12 and >1.55 provided robust discriminatory value, consistent with previous studies [34,38,39]. While PLR showed limited diagnostic value, it may still hold prognostic importance in multivariate models [40].

This study provides preliminary insights into the inflammatory and hematological profiles associated with triple-negative breast cancer (TNBC) in a Senegalese population. TNBC cases in this cohort were characterized by a systemic inflammatory and immunosuppressive profile, including elevated levels of neutrophils, CRP, NLR, and MLR, along with reduced lymphocyte and basophil percentages. These immune alterations may contribute to the aggressive clinical behavior of TNBC. Among the markers assessed, NLR demonstrated the most consistent ability to differentiate TNBC patients from healthy individuals, suggesting its potential value as a biological indicator of cancer-related inflammation.

However, these findings must be interpreted with caution. The cross-sectional, single-center design limits causal inference and precludes any assessment of prognostic or diagnostic utility. The relatively small sample size, absence of external validation, and lack of outcome-based analyses further restrict the generalizability and clinical applicability of the results. Additionally, baseline differences between the study groups, particularly between cancer patients and healthy controls, such as demographic, clinical, or metabolic variability, may have influenced the inflammatory markers observed. While this may introduce potential confounding, the findings still offer valuable exploratory insights into immune-inflammatory patterns associated with TNBC in this population.

As such, this study should be viewed as an exploratory biological characterization and a basis for hypothesis generation. Future research should involve larger, multicenter, and prospective cohorts with longitudinal follow-up to validate these observations and determine their potential relevance in clinical practice.

## 5. Conclusions

This study contributes to a better understanding of triple-negative breast cancer by highlighting distinct hematological and inflammatory features observed in patients at the time of diagnosis. While these findings are not intended for diagnostic or therapeutic use, they provide complementary information that may help refine the clinical and biological characterization of this aggressive subtype within our context. Further studies with larger cohorts are warranted to validate these observations and to explore their potential biological and clinical significance.

## Figures and Tables

**Table 1:** Sociodemographic, anthropometric, reproductive, lifestyle, and tumor characteristics of the study population.

Variable	TNBC (*n* = 40)	HDBC (*n* = 40)	Healthy Controls (*n* = 40)	*p* (TNBC vs. HDBC)	*p* (TNBC vs. Healthy Controls)
**SOCIODEMOGRAPHIC CHARACTERISTICS**					
**Age (years)**					
	Mean ± SD: 46.03 ± 13.52 Median [IQR]: 45.5 [39–54.75]	Mean ± SD: 48.78 ± 12.93 Median [IQR]: 47.5 [40.25–56.75]	Mean ± SD: 49.65 ± 8.27 Median [IQR]: 48.0 [44.25–55.75]	-	-
**ANTHROPOMETRIC CHARACTERISTICS**					
**BMI (kg/m^2^)**					
	Mean ± SD: 24.00 ± 6.31 Median [IQR]: 23.6 [19.48–26.71]	Mean ± SD: 24.46 ± 5.31 Median [IQR]: 23.56 [21.21–26.69]	Mean ± SD: 27.82 ± 8.25 Median [IQR]: 27.09 [24.08–29.41]	0.568	0.003 *
BMI Categories	Underweight: 8 (20.0%) Normal: 19 (47.5%) Overweight: 9 (22.5%) Obese: 4 (10.0%)	Underweight: 3 (7.5%) Normal: 25 (62.5%) Overweight: 6 (15.0%) Obese: 6 (15.0%)	Underweight: 1 (2.5%) Normal: 12 (30.0%) Overweight: 21 (52.5%) Obese: 6 (15.0%)	0.150	0.047 *
**GYNECO-OBSTETRIC CHARACTERISTICS**					
**Age at Menarche (years)**					
	Mean ± SD: 14.15 ± 1.88 Median [IQR]: 14.0 [13–15]	Mean ± SD: 14.45 ± 1.78 Median [IQR]: 14.0 [14–15]	Mean ± SD: 14.15 ± 1.85 Median [IQR]: 14.0 [13–15]	0.277	0.828
**Gravidity**					
	Mean ± SD: 5.13 ± 3.01 Median [IQR]: 5.0 [3–5]	Mean ± SD: 4.00 ± 2.93 Median [IQR]: 4.0 [1–5]	Mean ± SD: 4.30 ± 3.42 Median [IQR]: 4.0 [1–5]	0.040 *	0.227
Gravidity Categories	Multigravida: 35 (87.5%) Primigravida: 4 (10.0%) Nulligravida: 1 (2.5%)	Multigravida: 31 (77.5%) Primigravida: 4 (10.0%) Nulligravida: 5 (12.5%)	Multigravida: 31 (77.5%) Primigravida: 2 (5.0%) Nulligravida: 7 (17.5%)	0.300	0.102
**Parity**					
	Mean ± SD: 4.18 ± 2.28 Median [IQR]: 4.0 [3–6.75]	Mean ± SD: 3.08 ± 2.73 Median [IQR]: 3.0 [2–5]	Mean ± SD: 3.10 ± 2.41 Median [IQR]: 3.0 [2–6]	0.014 *	0.026 *
Parity Categories	Multiparity: 34 (85.0%) Primiparity: 3 (7.5%) Nulliparity: 3 (7.5%)	Multiparity: 27 (67.5%) Primiparity: 5 (12.5%) Nulliparity: 8 (20.0%)	Multiparity: 29 (72.5%) Primiparity: 3 (7.5%) Nulliparity: 8 (20.0%)	0.141	0.362
**Contraceptive Use**					
	15 (37.5%)	13 (32.5%)	23 (57.5%)	0.791	0.057
**Menopausal Status**					
	19 (47.5%)	22 (55.0%)	26 (65.0%)	0.453	0.118
**Abortion History**					
	21 (52.5%)	25 (62.5%)	20 (50.0%)	0.523	1.000
**LIFESTYLE FACTORS**					
Sedentary Lifestyle	33 (82.5%)	37 (92.5%)	38 (95.0%)	0.289	0.180
Smoking	0 (0%)	0 (0%)	0 (0%)	-	-
Alcohol Consumption	0 (0%)	0 (0%)	1 (2.5%)	-	-
**FAMILY HISTORY OF CANCER**					
Family History of Cancer	12 (30.0%)	9 (22.5%)	15 (37.5%)	0.607	0.664
**COMORBIDITIES**					
Diabetes	2 (5.0%)	5 (12.5%)	0 (0%)	0.453	-
Hypertension (HTA)	10 (25.0%)	8 (20.0%)	0 (0%)	0.804	-
**TUMOR CHARACTERISTICS**					
T Stage	T4: 30 (75.0%) T3: 7 (17.5%) T2: 2 (5.0%) T1: 1 (2.5%)	T4: 24 (60.0%) T3: 8 (20.0%) T2: 7 (17.5%) T1: 1 (2.5%)	-	0.311	-
N Stage	N0: 2 (5.0%) N1: 25 (62.5%) N2: 9 (22.5%) N3: 4 (10.0%)	N0: 7 (17.5%) N1: 22 (55.0%) N2: 8 (20.0%) N3: 3 (7.5%)	-	0.597	-
Clinical Stage	III: 25 (62.5%) IV: 13 (32.5%) II: 2 (5.0%)	III: 21 (52.5%) IV: 12 (30.0%) II: 6 (15.0%) I: 1 (2.5%)	-	0.975	-
SBR Grade	III: 18 (45.0%) II: 21 (52.5%) I: 1 (2.5%)	III: 9 (22.5%) II: 25 (62.5%) I: 6 (15.0%)	-	0.112	-
Ki67 Expression	≥10%: 35 (87.5%) <10%: 5 (12.5%)	≥10%: 40 (100%)	-	-	-

Abbreviations: TNBC = triple-negative breast cancer; HDBC = hormone-dependent breast cancer; SD = standard deviation; IQR = interquartile range; HTA = hypertension. Continuous variables were analyzed using non-parametric tests (median comparisons). Categorical variables were analyzed using chi-square tests for overall association. * Statistically significant differences (*p* < 0.05).

**Table 2:** Hematological parameters in TNBC, HDBC, and healthy control groups.

Hematological Parameters	TNBC Median (IQR)	HDBC Median (IQR)	Healthy Controls Median (IQR)	*p*
Red blood cells (×10^6^/µL)	4.47 (3.96–4.84)	4.65 (4.25–4.83)	4.49 (4.13–4.76)	0.397
Hemoglobin (g/dL)	12.20 (11.20–13.27)	13.02 (11.67–13.77)	12.75 (11.90–13.20)	0.339
Hematocrit (%)	38.00 (35.85–39.22)	39.80 (35.12–41.67)	38.95 (37.15–40.50)	0.738
MCV (fL)	83.80 (79.00–89.00)	85.09 (81.27–88.27)	87.70 (83.50–90.92)	0.025
MCH (pg)	27.15 (24.65–28.57)	27.97 (26.87–29.00)	28.45 (27.70–29.47)	0.046
MCHC (g/dL)	32.00 (31.10–33.00)	32.82 (31.90–33.77)	32.35 (31.60–32.85)	0.286
Platelets (×10^3^/µL)	299.50 (268.00–376.00)	272.00 (240.50–315.00)	298.50 (267.00–351.75)	0.098
White blood cells (×10^3^/µL)	5.34 (4.14–6.09)	5.49 (4.65–6.69)	4.54 (4.11–5.55)	0.018
Neutrophils (×10^3^/µL)	3.52 (2.91–3.98)	2.85 (2.34–3.60)	1.88 (1.60–2.50)	<0.001
Neutrophils (%)	56.90 (53.72–60.52)	50.70 (45.62–62.22)	43.05 (37.15–51.10)	<0.001
Eosinophils (×10^3^/µL)	0.11 (0.04–0.14)	0.11 (0.03–0.16)	0.14 (0.07–0.20)	0.789
Eosinophils (%)	2.00 (0.90–2.80)	1.60 (0.90–3.10)	3.00 (1.50–3.70)	0.019
Basophils (×10^3^/µL)	0.02 (0.02–0.04)	0.04 (0.03–0.06)	0.03 (0.02–0.05)	0.019
Basophils (%)	0.50 (0.40–0.60)	0.70 (0.40–1.10)	0.70 (0.50–0.90)	0.017
Lymphocytes (×10^3^/µL)	1.77 (1.45–2.57)	1.82 (1.51–2.19)	2.09 (1.68–2.69)	0.301
Lymphocytes (%)	32.50 (25.90–34.10)	34.75 (26.07–38.80)	45.25 (37.40–50.87)	<0.001 *
Monocytes (×10^3^/µL)	0.42 (0.36–0.67)	0.48 (0.39–0.64)	0.35 (0.31–0.51)	0.017
Monocytes (%)	7.95 (6.50–9.20)	8.45 (7.02–11.30)	7.70 (6.70–9.15)	0.373

(*) Repeated-measures ANOVA test. TNBC = triple-negative breast cancer; HDBC = hormone receptor-positive breast cancer; Median (IQR) = median (interquartile range). Values with *p* < 0.05 are considered statistically significant.

**Table 3:** Predictive value of inflammatory markers (NLR, MLR, and PLR) for distinguishing TNBC from HDBC and healthy controls based on ROC curve analysis.

	Cut-Off	Se	Sp	AUC	95% CI	*p*
TNBC vs. HDBC						
NLR	1.55	65%	60%	0.572	0.444–0.700	0.268
MLR	0.24	50%	42.5%	0.444	0.317–0.572	0.392
PLR	144.73	52.5%	55%	0.518	0.390–0.646	0.780
TNBC vs. Healthy controls						
NLR	1.12	90%	65%	0.847	0.762–0.933	0.000 *
MLR	0.20	62.5%	67.5%	0.684	0.566–0.801	0.005 *
PLR	136.50	65%	45%	0.563	0.436–0.691	0.331

Abbreviations: Se = sensitivity, Sp = specificity, AUC = area under the curve, CI = confidence interval. * Statistically significant (*p* < 0.05).

**Table 4:** Adjusted odds ratios for hematological and inflammatory parameters associated with TNBC compared to HDBC and healthy controls.

Group	Parameter	Adjusted OR	95% CI	*p*
TNBC vs. HDBC				
	Neutrophils (×10^3^/µL)	1.61	[1.04–2.50]	0.034
	Inflammation *	5.00	[1.45–17.27]	0.011
	MCH	0.62	[0.40–0.97]	0.036
	Basophils (%)	0.30	[0.09–0.92]	0.035
	MLR > 0.24	0.21	[0.047–0.91]	0.038
	NLR > 1.55	5.08	[1.41–18.28]	0.013
TNBC vs. Healthy controls				
	Neutrophils (×10^3^/µL)	12.21	[2.00–74.48]	0.007
	MCH	0.74	[0.58–0.95]	0.015
	Lymphocytes (%)	0.85	[0.78–0.93]	0.001
	White Blood Cells	0.19	[0.05–0.77]	0.020
	Neutropenia	0.14	[0.04–0.48]	0.002
	Inflammation	4.92	[1.46–16.59]	0.010
	NLR	26.43	[3.33–209.66]	0.002
	NLR > 1.12	11.5	[2.71–48.77]	0.001
	PLR	0.98	[0.95–1.00]	0.053
	CRP	1.02	[1.002–1.04]	0.026

* Inflammation defined as CRP > 6 mg/L.

## Data Availability

The dataset supporting this study is available in Mendeley Data [41]. See Supplementary Material.

## Supplementary Materials

The following supporting information can be downloaded at https://www.mdpi.com/article/10.3390/diagnostics16030494/s1, Table S1: Data.

## Abbreviations

The following abbreviations are used in this manuscript:

TNBC	Triple-Negative Breast Cancer
HDBC	Hormone-Dependent Breast Cancer
NLR	Neutrophil-to-Lymphocyte Ratio
MLR	Monocyte-to-Lymphocyte Ratio
PLR	Platelet-to-Lymphocyte Ratio
CRP	C-Reactive Protein
CBC	Complete Blood Count
IHC	Immunohistochemistry
ER	Estrogen Receptor
PR	Progesterone Receptor
HER2	Human Epidermal Growth Factor Receptor 2
Ki-67	Proliferation Marker Ki-67
ROC	Receiver Operating Characteristic
AUC	Area Under the Curve
OR	Odds Ratio
HTA	Hypertension
WBC	White Blood Cell
MCV	Mean Corpuscular Volume
MCH	Mean Corpuscular Hemoglobin
MCHC	Mean Corpuscular Hemoglobin Concentration
BI-RADS	Breast Imaging Reporting and Data System
TAM	Tumor-Associated Macrophage
ISH	In Situ Hybridization
ASCO/CAP	American Society of Clinical Oncology/College of American Pathologists

## References

[1] Vista do Impacto do Fenótipo Triplo-Negativo no Prognóstico de Pacientes com Câncer de Mama de uma Unidade de Referência no Brasil Central. https://revistamastology.emnuvens.com.br/rbm/article/view/57/45.

[2] Wahba H.A., El-Hadaad H.A. (2015). Current approaches in treatment of triple-negative breast cancer. Cancer Biol. Med..

[3] Koh C.H., Bhoo-Pathy N., Ng K.L., Jabir R.S., Tan G.H., See M.H., Jamaris S., Taib N.A. (2015). Utility of pre-treatment neutrophil-lymphocyte ratio and platelet-lymphocyte ratio as prognostic factors in breast cancer. Br. J. Cancer.

[4] Pistelli M., De Lisa M., Ballatore Z., Caramanti M., Pagliacci A., Battelli N., Ridolfi F., Santoni M., Maccaroni E., Bracci R. (2015). Pre-treatment neutrophil to lymphocyte ratio may be a useful tool in predicting survival in early triple negative breast cancer patients. BMC Cancer.

[5] Huszno J., Kolosza Z. (2019). Prognostic value of the neutrophil-lymphocyte, platelet-lymphocyte and monocyte-lymphocyte ratio in breast cancer patients. Oncol. Lett..

[6] Bakker N.A.M., Garner H., van Dyk E., Champanhet E., Klaver C., Duijst M., Voorwerk L., Nederlof I., Voorthuis R., Liefaard M.C. (2025). Triple-negative breast cancer modifies the systemic immune landscape and alters neutrophil functionality. npj Breast Cancer.

[7] Alizamir A., Azad S.D., Pirdehghan A., Moradi A. (2022). Preoperative Neutrophil: Lymphocyte Ratio, Platelet: Lymphocyte Ratio, and C-Reactive Protein Levels Predictive Value in Determining the Severity of Breast Mass. Iran. J. Pathol..

[8] Corbeau I., Jacot W., Guiu S. (2020). Neutrophil to Lymphocyte Ratio as Prognostic and Predictive Factor in Breast Cancer Patients: A Systematic Review. Cancers.

[9] Oshi M., Newman S., Tokumaru Y., Yan L., Matsuyama R., Endo I., Takabe K. (2020). Inflammation Is Associated with Worse Outcome in the Whole Cohort but with Better Outcome in Triple-Negative Subtype of Breast Cancer Patients. J. Immunol. Res..

[10] Liu C., Huang Z., Wang Q.S., Sun B., Ding L.J., Meng X.Y., Wu S. (2016). Usefulness of neutrophil-to-lymphocyte ratio and platelet-to-lymphocyte ratio in hormone-receptor-negative breast cancer. Onco Targets Ther..

[11] Boaro C.M., Diefenthäeler L.M., Costa GKda Tiscoski K.A., Alves R.J.V., Berto M.D., Bica C.G. (2022). Hematological ratios as prognostic indicators in patients with triple-negative breast cancer in southern Brazil. Mastology.

[12] Jia W., Wu J., Jia H., Yang Y., Zhang X., Chen K., Su F. (2015). The Peripheral Blood Neutrophil-To-Lymphocyte Ratio Is Superior to the Lymphocyte-To-Monocyte Ratio for Predicting the Long-Term Survival of Triple-Negative Breast Cancer Patients. PLoS ONE.

[13] Azab B., Amundson J.R., Cioci A., Stuart H., Yakoub D., Avisar E., Moffat F., Livingstone A.S., Franceschi D. (2021). The Usefulness of the Pretreatment Neutrophil/Lymphocyte Ratio as a Predictor of the 5-Year Survival in Stage 1-3 Triple Negative Breast Cancer Patients. Breast Care.

[14] Guo W., Lu X., Liu Q., Zhang T., Li P., Qiao W., Deng M. (2019). Prognostic value of neutrophil-to-lymphocyte ratio and platelet-to-lymphocyte ratio for breast cancer patients: An updated meta-analysis of 17079 individuals. Cancer Med..

[15] Wang C., Shi Q., Zhang G., Wu X., Yan W., Wan A., Xiong S., Yuan L., Tian H., Ma D. (2024). Two Hematological Markers Predicting the Efficacy and Prognosis of Neoadjuvant Chemotherapy Using Lobaplatin Against Triple-Negative Breast Cancer. Oncologist.

[16] Chae S., Kang K.M., Kim H.J., Kang E., Park S.Y., Kim J.H., Kim S.H., Kim S.W., Kim E.K. (2018). Neutrophil-lymphocyte ratio predicts response to chemotherapy in triple-negative breast cancer. Curr. Oncol..

[17] Pathology Outlines—Hormone Receptors. https://www.pathologyoutlines.com/topic/breastmalignanthormonereceptors.html.

[18] Gaye P.M., Diouf M., Ba M.B., Diouf D., Sarr F.N., Mané M., Sarr M., Dieng M.M., Ka S., Dem A. (2021). Epidemiological, Diagnosis, Therapeutic and Evolving Profile of Triple Negative Breast Cancer in Senegal. Adv. Breast Cancer Res..

[19] Ndiaye M., Dieng S., Thiam J., Diallo A.C., Diouf D., Ka S., Dem A. (2021). Evaluation des facteurs pronostiques dans le cancer du sein chez la femme au Sénégal: à propos de 288 cas. Afr. J. Oncol..

[20] Jiagge E., Jibril A.S., Davis M., Murga-Zamalloa C., Kleer C.G., Gyan K., Divine G., Hoenerhoff M., Bensenhave J., Awuah B. (2018). Androgen Receptor and ALDH1 Expression Among Internationally Diverse Patient Populations. J. Glob. Oncol..

[21] Prakash O., Hossain F., Danos D., Lassak A., Scribner R., Miele L. (2020). Racial Disparities in Triple Negative Breast Cancer: A Review of the Role of Biologic and Non-biologic Factors. Front. Public Health.

[22] Martini R., Delpe P., Chu T.R., Arora K., Lord B., Verma A., Bedi D., Karanam B., Elhussin I., Chen Y. (2022). African Ancestry-Associated Gene Expression Profiles in Triple-Negative Breast Cancer Underlie Altered Tumor Biology and Clinical Outcome in Women of African Descent. Cancer Discov..

[23] Nwagu G.C., Bhattarai S., Swahn M., Ahmed S., Aneja R. (2021). Prevalence and Mortality of Triple-Negative Breast Cancer in West Africa: Biologic and Sociocultural Factors. JCO Glob. Oncol..

[24] Zheng C., Xu X., Wu M., Xue L., Zhu J., Xia H., Ding S., Fu S., Wang X., Wang Y. (2023). Neutrophils in triple-negative breast cancer: An underestimated player with increasingly recognized importance. Breast Cancer Res..

[25] Madhu Y., Jain S., Jain P., Kashyap N., Mangalhara K.C., Prakash Jain B. (2025). Hematological and Biochemical Serum Markers in Breast Cancer: Diagnostic, Therapeutic, and Prognostic Significance. Explor. Res. Hypothesis Med..

[26] Allin K.H., Nordestgaard B.G., Flyger H., Bojesen S.E. (2011). Elevated pre-treatment levels of plasma C-reactive protein are associated with poor prognosis after breast cancer: A cohort study. Breast Cancer Res..

[27] Wei B., Yao M., Xing C., Wang W., Yao J., Hong Y., Liu Y., Fu P. (2016). The neutrophil lymphocyte ratio is associated with breast cancer prognosis: An updated systematic review and meta-analysis. Onco Targets Ther..

[28] Inoue Y., Fujishima M., Ono M., Masuda J., Ozaki Y., Maeda T., Uehiro N., Takahashi Y., Kobayashi T., Sakai T. (2022). Clinical significance of the neutrophil-to-lymphocyte ratio in oligometastatic breast cancer. Breast Cancer Res. Treat..

[29] Eo W.K., Jeong D.W., Chang H.J., Won K.Y., Choi S.I., Kim S.H., Chun S.W., Oh Y.L., Lee T.H., Kim Y.O. (2015). Absolute monocyte and lymphocyte count prognostic score for patients with gastric cancer. World J. Gastroenterol..

[30] Zhou X., Du Y., Huang Z., Xu J., Qiu T., Wang J., Wang T., Zhu W., Liu P. (2014). Prognostic value of PLR in various cancers: A meta-analysis. PLoS ONE.

[31] Krenn-Pilko S., Langsenlehner U., Thurner E.M., Stojakovic T., Pichler M., Gerger A., Kapp K.S., Langsenlehner T. (2014). The elevated preoperative platelet-to-lymphocyte ratio predicts poor prognosis in breast cancer patients. Br. J. Cancer.

[32] Zhang M., Huang X.Z., Song Y.X., Gao P., Sun J.X., Wang Z.N. (2017). High Platelet-to-Lymphocyte Ratio Predicts Poor Prognosis and Clinicopathological Characteristics in Patients with Breast Cancer: A Meta-Analysis. BioMed Res. Int..

[33] Hu Y., Wang S., Ding N., Li N., Huang J., Xiao Z. (2020). Platelet/Lymphocyte Ratio Is Superior to Neutrophil/Lymphocyte Ratio as a Predictor of Chemotherapy Response and Disease-free Survival in Luminal B-like (HER_2_^−^) Breast Cancer. Clin. Breast Cancer.

[34] Chen F., Chen D., Jin L., Xu C., Zhao W., Hu W. (2022). Prognostic Significance of Neutrophil-to-Lymphocyte Ratio and C-Reactive Protein/Albumin Ratio in Luminal Breast Cancers With HER2-Negativity. Front. Oncol..

[35] Zhu J., Cheng J., Ma Y., Wang Y., Zou Z., Wang W., Shi H., Meng Y. (2025). The value of inflammation-related indicators in chemotherapy efficacy and disease-free survival of triple-negative breast cancer. Eur. J. Med. Res..

[36] von Au A., Shencoru S., Uhlmann L., Mayer L., Michel L., Wallwiener M., Hennigs A., Deutsch T., Riedel F., Heil J. (2023). Predictive value of neutrophil-to-lymphocyte-ratio in neoadjuvant-treated patients with breast cancer. Arch. Gynecol. Obstet..

[37] Shi K., Westhuyzen J., Gortman A., Shakespeare T.P., Aherne N.J. (2022). Prognostic Value of the Neutrophil-Lymphocyte Ratio in Triple Negative Breast Cancer Patients. Ann. Clin. Lab. Sci..

[38] Bae S.J., Cha Y.J., Yoon C., Kim D., Lee J., Park S., Cha C., Kim J.Y., Ahn S.G., Park H.S. (2020). Prognostic value of neutrophil-to-lymphocyte ratio in human epidermal growth factor receptor 2-negative breast cancer patients who received neoadjuvant chemotherapy. Sci. Rep..

[39] Yang Z., Zhang Y., Song M., Huang X., Lin Y., Yang H. (2023). The interaction between systemic inflammatory markers and polygenic risk score in breast cancer risk: A cohort study in the UK Biobank. Cancer Epidemiol..

[40] Zhao Z., Xu H., Ma B., Dong C. (2025). Prognostic value of platelet to lymphocyte ratio (PLR) in breast cancer patients receiving neoadjuvant therapy: A systematic review and meta-analysis. Front. Immunol..

[41] Barry N.O.K. (2025). Systemic Hematological and Inflammatory Profiles in Triple-Negative Breast Cancer in a Senegalese Cohort, Mendeley Data, V1. https://data.mendeley.com/datasets/ck5gfhvbgz/1.

